# Efficacy of Vaporized Hydrogen Peroxide Combined with Silver Ions against Multidrug-Resistant Gram-Negative and Gram-Positive Clinical Isolates

**DOI:** 10.3390/ijms232415826

**Published:** 2022-12-13

**Authors:** Sandra Patricia Rivera-Sánchez, José María Rojas-Abadía, John Jairo Ríos-Acevedo, Ana Fernanda Mejía-Hurtado, Luz Natalia Espinosa-Moya, Iván Darío Ocampo-Ibáñez

**Affiliations:** 1 Research Group of Microbiology, Industry and Environment (GIMIA), Faculty of Basic Sciences, Universidad Santiago de Cali, Cali 760035, Colombia; 2 Laboratorio de Salud Pública Departamental, Secretaria Departamental de Salud del Valle del Cauca, Gobernación del Valle del Cauca, Cali 760045, Colombia; 3 Research Group of Electrochemistry and Environment (GIEMA), Faculty of Basic Sciences, Universidad Santiago de Cali, Cali 760035, Colombia; 4 Department of Public Health and Community Medicine, Universidad ICESI, Cali 760032, Colombia

**Keywords:** multidrug-resistant *Klebsiella pneumoniae*, multidrug-resistant *Pseudomonas aeruginosa*, methicillin-resistant *Staphylococcus aureus*, hydrogen peroxide with silver ions

## Abstract

Antimicrobial resistance (AMR) is a serious public health problem that results in high morbidity and mortality rates. In particular, multidrug-resistant (MDR) strains circulating in hospital settings pose a major threat as they are associated with serious nosocomial infections. Therefore, regular cleaning and disinfection procedures, usually using chemical disinfectants, must be implemented in these facilities. Hydrogen peroxide (HP)-based disinfectants have proven high microbicidal activity and several comparative advantages over conventional disinfectants. We assessed the in vitro biocidal activity of an 8% HP solution combined with 30 mg/L silver ions (HP + Ag) against MDR clinical isolates of *Klebsiella pneumoniae* (MDRKp) and *Pseudomonas aeruginosa* (MDRPa), and methicillin-resistant Staphylococcus aureus (MRSA). Accordingly, the in vitro antibacterial activity was determined using the macrodilution method, and the efficacy was determined for 30 min in terms of (1) activity on bacteria in suspension and (2) activity on surfaces using vaporized HP + Ag on a 20 cm^2^ stainless steel surface. A strong bactericidal effect of HP + Ag was observed against MDRKp, MDRPa, and MRSA strains, with minimum inhibitory concentrations and minimum bactericidal concentrations between 362.5 and 5800 mg/L. A strong effect was observed during the 30 min of HP + Ag exposure to the resistant clinical isolates, with over 4-Log_10_ reduction in CFUs. Regarding the efficacy of the disinfectant on surfaces, bacterial load reductions of >99% were observed. These results suggest that HP + Ag is potentially useful as an effective disinfectant for decontaminating surfaces in hospital settings suspected of contamination with MDR bacteria.

## 1. Introduction

Antimicrobial resistance (AMR) to conventional antimicrobials is a serious public health problem, resulting in ineffective empirical antimicrobial therapy in the treatment of bacterial infections [1]. AMR leads to increased morbidity and mortality rates and higher medical costs [2]. Excessive or inappropriate antibiotic use, self-medication, and infections in healthcare facilities are factors that contribute significantly to the emergence of resistant bacterial strains [1]. In view of the concern regarding AMR and its serious impact on global public health, the World Health Organization has recently published a list of bacteria that pose the greatest threat to human health [3]. Multidrug-resistant (MDR) bacteria are the most critical group of all bacteria. They include gram-negative bacteria, such as MDR *Klebsiella pneumoniae* and *Pseudomonas aeruginosa*, and gram-positive bacteria, such as methicillin-resistant *Staphylococcus aureus* (MRSA) and vancomycin-resistant *Staphylococcus aureus* [3]. These bacteria have spread worldwide, and MDR strains have been found circulating in both community and hospital settings [4,5,6,7,8]. In particular, resistant bacteria can cause nosocomial infections in the latter, especially among patients requiring devices such as ventilators and venous catheters [3,4,5,6,7]. Common infections associated with resistant strains include life-threatening infections such as pneumonia, bloodstream infections, urinary tract infections, wound infections, and meningitis [3,8,9,10]. The incidence of nosocomial infections caused by this type of bacteria is high. For example, among hospitalized patients in the United States in 2017, there were (1) 13,100 estimated infections and 1100 deaths due to carbapenem-resistant Enterobacterales, including *K. pneumoniae*; (2) 32,600 infections and 2700 deaths due to MDR *P. aeruginosa* (MDRPa); and (3) 323,700 infections and 10,600 deaths due to MRSA [9,10]. In Colombia, strains of MDR *K. pneumoniae* (MDRKp) and MDRPa, whose resistance is mediated by *bla_KPC_*, *bla_VIM_*, *bla_IMP_*, *bla_NDM_*, and *bla_OXA-48_*, have been found circulating in both community and hospital settings [11,12,13,14]. Resistant bacterial clinical isolates and MDR bacteria bearing this wide diversity of genes have been reported to be responsible for nosocomial infections in healthcare institutions in Colombia. In 2020, 5256 cases of device-associated infections in intensive care units (ICUs) and 504 cases of infections associated with surgical procedures were reported in this country [15].

Different surfaces and equipment (side rails, stethoscopes, medical records, ultrasound machines, etc.) in ICUs, operating rooms, hematology and oncology units, among other hospital areas, may often be contaminated with MDR clinical isolates [8,16]. Bacteria can survive for up to several months on these dry surfaces, and therefore nosocomial infections can occur due to cross-transmission from healthcare professionals or via direct contact of the patient with the colonized surface [16]. The implementation of periodic cleaning and disinfection procedures is necessary to eliminate or reduce the load of resistant pathogenic microorganisms on surfaces and equipment [16]. The CDC Guideline for Disinfection and Sterilization in Healthcare Facilities recommends a wide variety of chemical compounds for surfaces or equipment [17]. Commonly used compounds for disinfecting and sterilizing these facilities include (1) alcohols, e.g., water-soluble chemical compounds such as ethyl alcohol and isopropyl alcohol; (2) chlorine and chlorine-containing compounds such as liquid hypochlorites (e.g., sodium hypochlorite) or solid hypochlorites (e.g., calcium hypochlorite); (3) liquid or gaseous formaldehyde; (4) glutaraldehyde; (5) ortho-phthalaldehyde (OPA); (6) quaternary ammonium compounds; (7) phenolics; (8) iodophors; and (9) hydrogen peroxide (HP) [17,18]. Comparative disadvantages of some of these compounds include the limited spectrum of microorganisms they can control, damage to equipment from prolonged use, and adverse effects on human health and the environment [17].

In particular, HP is a chemical disinfectant that can be used in several environmental matrices, including air, water, wastewater, surfaces, and soil, among others [19], and is widely used for the disinfection and sterilization of surfaces and equipment in healthcare facilities [17,19,20]. In these settings, disinfection with HP has many advantages over those with conventional compounds such as aldehyde-based disinfectants (glutaraldehyde and OPA) or chlorine, some of which include potent broad-spectrum antimicrobial activity, little damage to delicate surfaces or equipment, good stability in the environment, no associated toxicity, and reduced environmental pollution [17,19,20]. HP has been shown to exhibit good microbicidal activity, even against a wide variety of bacterial species resistant to conventional antibiotics, as it functions by releasing destructive oxygen and hydroxyl-free radicals that cause damage to membrane lipids, DNA, and other essential cellular components [17,20]. HP is a highly versatile disinfectant as it can be used in liquid, gaseous, or vaporized form as well as in combination with other agents (e.g., adjuvants, excipients, or synergistic chemicals) to enhance its effect [17,20]. Vaporized hydrogen peroxide (VHP) is widely used for surface disinfection in the pharmaceutical industry and healthcare facilities [20,21], and it may be combined with different concentrations of silver ions. Silver acts as an adjuvant and bacteriostatic agent on surfaces once the peroxide evaporates [20]. VHP is an effective bactericide, even against resistant and MDR strains [19]. Both VHP [22,23] and VHP combined with silver ions [24] have been successfully used to decontaminate hospital units, especially ICUs, by removing MDR strain deposits. Nevertheless, the efficacy of these biocides depends on contact time and chemical concentration [19]. This study aimed to assess the in vitro activity of 8% VHP combined with 30 mg/L silver ions (VHP + Ag) on contaminated surfaces. The biocidal efficacy of this formulation was evaluated against MDRKp, MDRPa, and MRSA clinical isolates.

## 2. Results and Discussion

### 2.1. Resistance and Susceptibility of Clinical Bacterial Isolates

Table 1 and Table 2 show the antibiotic susceptibility and resistance profiles of the clinical isolates of *K. pneumoniae*, *P. aeruginosa*, and *S. aureus,* and ATCC strains. The *K. pneumoniae* isolates were classified as MDR strains based on their resistance to all categories of antimicrobials evaluated, including penicillin + β-lactamase inhibitors, cephamycins, extended-spectrum cephalosporins, carbapenems, aminoglycosides, and fluoroquinolones (Table 1). In addition, the presence of *bla_KPC_* for the production of class A serine carbapenemase was determined in these strains by confirmation of the molecular classes of carbapenemases and the Carba NP test (Table 1). The three strains of *P. aeruginosa* evaluated were all MDR, showing resistance to penicillin + β-lactamase inhibitors, antipseudomonal cephalosporins, carbapenems, aminoglycosides, fluoroquinolones, and polymyxins (Table 1). According to the molecular characterization, two MDRPa clinical isolates exclusively carried *bla_VIM_* and/or *bla_IMP_* encoding metallo-β-lactamase (MBL) enzymes (Table 1). Only one of the strains carried a combination of *bla_KPC_*, *bla_VIM_*, and/or *bla_IMP_* and *bla_NDM_*, indicating co-production of class A serine carbapenemase and MBL enzymes (Table 1). Based on these results, the MDRKp and MDRPa strains evaluated could be considered “high-risk clones” with resistance profiles similar to those endemic to Colombia [12,14,25,26]. Such clinical isolates have been found in hospital units, especially in ICUs, and therefore pose a serious threat to public health [12,14,25,26]. Although nosocomial transmission of Enterobacterales is usually person to person [27], resistant strains of *K. pneumoniae* carrying *bla_KPC_* have been previously found on different hospital surfaces and medical devices, including floors, walls, beds, stethoscopes, ventilators, and oxygen masks, among others [28]. Similarly, carbapenemase-producing *P. aeruginosa* strains carrying *bla_VIM_* have been found in hospital water-related reservoirs, such as water samples, drains, sinks, faucets, shower accessories, and toilet bowls [27]. These gram-negative bacterial species are able to colonize these environments, form biofilms, spread, and cause infections through direct or indirect contact with patients [27,29].

All three *S. aureus* clinical isolates showed resistance to antistaphylococcal penicillins, such as oxacillin, and were thus classified as MRSA strains (Table 2). Additionally, one of the MRSA strains was resistant to macrolides, specifically to erythromycin (Table 2). The MRSA strains included in this study showed resistance profiles similar to those of clinical isolates circulating in hospital settings in Colombia [30,31]. *S. aureus*, including MRSA strains, has been reported to be one of the leading causes of nosocomial infections worldwide, especially among patients in surgery, undergoing hemodialysis, and in ICU units, because of the long-term use of venous and urinary catheters [32,33,34]. The surfaces of some items in these units, such as beds, operating tables, infusion pumps, and electrocardiography devices, among others, have been found to be contaminated with MRSA strains, which could lead to cross-contamination via the hands of healthcare personnel [34,35,36].

### 2.2. In Vitro Antibacterial Activity of HP + Ag against MDR Clinical Isolates

The bactericidal activity of HP and HP + Ag has been previously studied owing to their high killing power on bacterial cells [19,24,37,38]. Table 3 summarizes the in vitro antibacterial activity of 8% HP combined with 30 mg/L silver ions against MDRKp, MDRPa, and MRSA clinical isolates. HP + Ag exhibited activity against gram-negative ATCC strains, such as *E. coli* and *P. aeruginosa*, with MIC values between 725 and 2900 mg/L (Table 3). Similarly, an effect against *S. aureus* strain ATCC 25923 was observed; however, the MIC value (363 mg/L) was lower than those found for gram-negative strains (Table 3). The efficacy of HP + Ag against gram-negative and gram-positive ATCC strains was in agreement with those reported in previous studies on HP and HP with silver ions against *P. aeruginosa*, *S. aureus*, and *E. coli* laboratory strains [24,39,40,41,42]. Similarly, all the resistant clinical isolates evaluated here were susceptible to HP + Ag, with MIC values between 362.5 and 5800 mg/L (MDRKp and MDRPa) and between 725 and 1450 mg/L (MRSA) (Table 3). Our results are in agreement with those previously reported for HP and HP with silver ions showing antimicrobial activity against resistant strains and MDR *Acinetobacter baumannii* [39,42,43], *K. pneumoniae* [39,43], *P. aeruginosa* [24,39,43], and *S. aureus* [24,39], with MIC values between 0.5 and 20 mM [43].

HP + Ag showed high activity against MDRKp, MDRPa, and MRSA isolates, with MIC values between 362.5 and 5800 mg/L, whereas MIC values for the control were between 2500 and 5500 mg/L (Table 3). When comparing the efficacy of HP + Ag and 5% hypochlorite against resistant clinical isolates, significant differences were observed for MDRKp and MRSA but not for MDRPa (Table 3). This indicates that HP + Ag was more effective than 5% hypochlorite, which is one of the most widely used disinfectants globally, healthcare facilities included [17,44].

Intraspecies comparisons for HP + Ag efficacy showed significant differences between ATCC strains susceptible to conventional antibiotics and resistant clinical isolates. In particular, significant differences were found between susceptible laboratory strains of the gram-positive *S. aureus* and clinical MRSA isolates (*S. aureus* ATCC 25923 and MRSA) (*p* = 0.03). These important differences could be attributed to resistance mechanisms that MRSA may have, which would modulate HP + Ag efficacy. Nevertheless, further research is needed to support this hypothesis. No significant differences were observed between strains of Enterobacterales (*E. coli* ATCC 25922 and MDRKp) (*p* = 0.07); however, MDR clinical isolates of *K. pneumoniae* were more susceptible to HP + Ag and showed the lowest MIC value (362.5 mg/L; Table 3). No significant differences were observed between *P. aeruginosa* ATCC 27853 and MDRPa (*p* = 0.21) when median MICs were compared. These results suggest that HP + Ag activity against *K. pneumoniae* and *P. aeruginosa* is not dependent on their phenotypic and genotypic characteristics related to their resistance pattern to conventional antibiotics. Therefore, this study proves the potential of HP + Ag as an alternative disinfectant for the control of MDR clinical isolates in hospital settings.

Finally, interspecies comparisons of resistant strains showed no significant differences between MRSA and MDRKp (*p* = 0.08) or between MRSA and MDRPa (*p* = 0.12). Based on these results, HP + Ag is active against resistant clinical isolates of both gram-negative and gram-positive bacterial species (Table 3). This is in agreement with the findings of previous studies showing the efficacy of solutions containing HP and silver ions against resistant strains of *K. pneumoniae, P. aeruginosa*, and *S. aureus* [24,39,42,43]. Significant differences were found between MICs for MDR *K. pneumoniae* and *P. aeruginosa* (*p* = 0.04), with high bactericidal activity of HP + Ag against MDRKp clinical isolates (Table 3). The significant difference in HP + Ag efficacy against MDRKp and MDRPa isolates can be related to the presence of virulence factors and resistance mechanisms in the strains evaluated (Table 1). In this regard, the capsule composed of polysaccharides, that contributes to the pathogenicity of some *Klebsiella pneumoniae* carbapenemase (KPC)-producing *K. pneumoniae* [45,46,47] and the mechanisms and systems associated with CST resistance in some strains of *P. aeruginosa* [5], could protect these bacteria from the antibacterial activity of HP + Ag by modulating its efficacy.

Because no differences were found between the MICs and MBCs of HP + Ag against all ATCC strains and resistant clinical isolates studied, this chemical disinfectant can be regarded as a bactericidal agent (Table 3). Our results demonstrate that HP + Ag is a chemical disinfectant with bactericidal activity against resistant clinical isolates of *K. pneumoniae*, *P. aeruginosa*, and *S. aureus*.

### 2.3. Antimicrobial Activity of HP + Ag on Bacteria in Suspension

**Figure 1:**
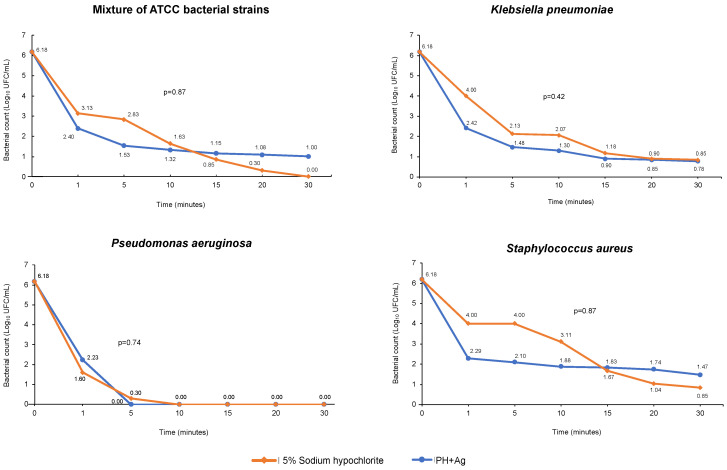
Death curves of the bacterial mixture of ATCC strains and resistant clinical isolates of *K. pneumoniae*, *P. aeruginosa*, and *S. aureus* exposed for 30 min to HP + Ag.

The disinfectant efficacy of HP + Ag on bacterial growth in suspension over time was tested using the MIC value of 1450 mg/L, which was the highest value among the medians found for all the strains evaluated. Efficacy was tested against a bacterial mixture of *E. coli* ATCC^®^ 25922™, *P. aeruginosa* ATCC^®^ 27853™, and *S. aureus* ATCC^®^ 25923™ strains, and against each resistant clinical isolate of *K. pneumoniae*, *P. aeruginosa*, and S. aureus at different times. Figure 1 shows the log reduction observed at different times of HP + Ag treatment. After 30 min of exposure, HP + Ag reduced the microbial load of all resistant clinical isolates to 4.71–6.18 Log_10_ CFU (Figure 1). However, no significant differences were observed when the reductions caused by HP + Ag and the control treatments were compared (Figure 1). Nevertheless, during the first 15 min of exposure, a higher susceptibility to HP + Ag was observed in both gram-negative and gram-positive resistant strains compared with 5% sodium hypochlorite (Figure 1). This result is consistent with that of a previous report on the persistence of MDR strain transmission after applying standard control measures, such as the routine use of sodium hypochlorite for daily cleaning and disinfection [19].

The bacterial death curves obtained during exposure to HP + Ag showed a remarkable variation in the Log_10_ CFU reduction at each time for the species evaluated (Figure 1). The antibacterial activity of HP + Ag against the mixture of ATCC strains was high and showed a 5.4 Log_10_ CFU reduction after 30 min of exposure (Figure 1). No significant differences were observed in the activity of HP + Ag against susceptible ATCC strains during the 30 min exposure compared with the control (Figure 1). This suggests that the disinfectant efficacy of the solution tested in this study is comparable to that of conventional disinfectants used in hospital settings, such as 5% hypochlorite. In addition, the bactericidal activity of HP + Ag against MDRKp strains was high, exhibiting a 5.4 Log_10_ CFU reduction during the 30 min exposure. It should be noted that approximately 70% of this reduction occurred during the first minute of contact (Figure 1). Our results are comparable with those of a previous study conducted by Watson et al. [41], who reported a 6 Log_10_ CFU reduction in KPC-producing *K. pneumoniae*; however, this was achieved after 50 min of exposure using only 35% HP. Another study assessed 5% HP combined with 0.1% silver ions against clinical isolates of MDR *K. pneumoniae* and reported a reduction close to 2 Log_10_ CFU after 35 min of exposure [39], which was much lower than that found in our study (Figure 1).

The bactericidal activity of HP + Ag against MDRPa clinical isolates over time was strong, with a 6.18 Log_10_ CFU reduction mostly within the first 5 min of exposure, making these the most susceptible strains. Thereafter, up to 30 min of contact, complete inactivation of the clinical isolates was observed, as no microorganisms survived in the samples exposed to HP + Ag (Figure 1). Previous studies determined the activity of HP and combinations of HP with silver ions against *P. aeruginosa* strains susceptible and resistant to conventional antibiotics [24,39,41]. Compared with our results, De Giglio et al. [24] reported a higher log reduction (8 Log_10_ CFU) between 5 and 30 min of exposure to 5% HP with 0.1% silver ions; however, they used susceptible ATCC strains of *P. aeruginosa*. Herruzo et al. [39] observed a reduction close to 4 Log_10_ CFU after exposing MDR *P. aeruginosa* strains to 5% HP with silver ions for 35 min. Similar to our findings, complete inactivation of extended-spectrum β-lactamase-producing *P. aeruginosa* strains was previously reported with a reduction of about 6 Log_10_ CFU [41]. However, in contrast to our findings, this reduction was reached after 100 min of exposure to a highly concentrated HP solution (35%) that did not contain silver ions [41].

The resistant clinical isolates of *S. aureus* also showed high susceptibility but lower than that of MDRKp and MDRPa isolates (Figure 1). Upon exposure to HP + Ag, a 4.44 Log_10_ CFU reduction was obtained for MRSA isolates; however, complete inactivation was not observed during the 30 min of contact (Figure 1). Similar reduction values (approximately 4 Log_10_ CFU) were previously reported using 5% HP with 0.1% silver ions [24] and 0.05% HP [42] against methicillin-resistant and MDR *S. aureus* clinical isolates for 30 min and 3 h, respectively. However, in our study, the reduction for MRSA was lower than that reported in a study that evaluated the activity of 35% HP for 100 min (6 Log_10_ CFU) [41]. 

### 2.4. Antimicrobial Activity of HP + Ag on Surfaces

To test the antimicrobial activity of HP + Ag on surfaces, 8% VHP combined with 30 mg/L silver ions was applied on two 20 cm^2^ stainless steel surfaces previously contaminated with a set of ATCC bacteria or with a set of resistant clinical isolates. VHP showed disinfectant activity on surfaces, as indicated by the inactivation of both ATCC bacteria and resistant clinical isolates (Table 4). A complete reduction in the load of ATCC strains was observed on the surface evaluated after exposure to VHP for 30 min (Table 4). Our results showed that VHP had activity against the set of resistant clinical isolates of *K. pneumoniae, P. aeruginosa*, and *S. aureus*; however, its efficacy was lower (99.81%) than that found for ATCC strains susceptible to conventional antibiotics (Table 4). These results demonstrate that VHP + Ag is useful for decontamination of surfaces containing bacteria susceptible and resistant to conventional antibiotics circulating in hospital settings.

Our results are in agreement with those of a previous study conducted by Davoudi et al. [38] on the antimicrobial activity of 0.3% VHP combined with 30 ppb silver ions on steel surfaces contaminated with clinical isolates of *E. coli*, *Proteus mirabilis*, and *K. pneumoniae* [38]. Their study showed that the disinfectant was highly effective in inactivating bacteria on steel surfaces after 15 min of exposure; however, susceptibility and/or resistance of clinical isolates to conventional antibiotics was not reported [38]. Another study also reported that a solution containing 5% HP and 0.1% silver ions showed activity against ATCC strains and MDR isolates of *S. aureus* and *P. aeruginosa* inoculated on steel surfaces after 10 and 15 min of exposure, respectively [24]. Reductions in microbial load on surfaces comparable to those found in our study have been reported previously [42,48]. Lemmen et al. [42] observed a reduction in bacterial load close to 100% on both porous (cotton) and nonporous (stainless steel) surfaces inoculated with MRSA strains upon exposure to 0.05% VHP. Rutala et al. [48] reduced the load of MRSA strains by 97% after spraying privacy curtains in ICUs with HP.

## 3. Materials and Methods

### 3.1. Selection, Identification, and Preparation of Clinical Isolates

Three clinical isolates were analyzed for each strain (MDRKp, MDRPa, and MRSA), yielding a total of nine clinical isolates. All isolates were obtained from clinical samples such as urine, secretions, and blood, which were collected at two tertiary hospitals in Cali, Colombia, between 2017 and 2019. The bacterial identity of the clinical isolates was confirmed at the Microbiology Laboratory of the Departmental Public Health Laboratory of Valle del Cauca (LSPD-Valle). Species were identified using the automated VITEK^®^ 2 system (bioMerieux, 9.02, Marcy l’Etoile, France), which uses established biochemical methods and substrates to assess carbon utilization, enzymatic activity, and resistance. The VITEK^®^ 2 GN ID card (Ref. 21341, bioMerieux, Marcy l’Etoile, France) was used to identify gram-negative species, and the VITEK^®^ 2 GP ID card (Ref 21342, bioMerieux, Marcy l’Etoile, France) was used to identify *S. aureus*.

### 3.2. Characterization of Clinical Isolates: Resistance Profiles

Resistance and susceptibility of clinical isolates to conventional antibiotics was determined via phenotypic and genotypic characterization. For the phenotypic characterization, the VITEK^®^ 2 Antimicrobial Susceptibility Testing N272 cards (VITEK^®^ AST-N272) (Ref. 414164, bioMerieux, Marcy l’Etoile, France) and VITEK^®^ 2 AST—P577 cards (Ref. 22218, bioMerieux, Marcy l’Etoile, France) were used for gram-negative and gram-positive isolates, respectively. The in vitro minimum inhibitory concentration (MIC) of each antibiotic was determined according to the clinical cutoff values defined by the Clinical Laboratory Standards Institute (CLSI) [49]. The antibiotic susceptibility of *K. pneumoniae* isolates was tested against amikacin (AMK), ampicillin/sulbactam (SAM), cefepime (FEP), cefoxitin (FOX), ceftazidime (CAZ), ceftriaxone (CRO), ciprofloxacin (CIP), doripenem (DOR), ertapenem (ETP), gentamicin (GEN), imipenem (IPM), meropenem (MEM), and piperacillin/tazobactam (TZP). The antibiotic susceptibility of *P. aeruginosa* isolates was tested against AMK, FEP, CAZ, CIP, DOR, GEN, IPM, MEM, TZP, and colistin (CST). The CST resistance of all MDRPa isolates was confirmed using the CLSI broth macrodilution reference method [50]. The antibiotic susceptibility of *S. aureus* isolates was tested against ampicillin, CIP, clindamycin, erythromycin, GEN, levofloxacin, linezolid, minocycline, moxifloxacin, nitrofurantoin, oxacillin, quinupristin/dalfopristin, rifampicin, teicoplanin, tetracycline, trimethoprim-sulfamethoxazole, and vancomycin. For gram-negative clinical isolates showing resistance to extended-spectrum cephalosporins and to at least one carbapenem, resistance mechanisms were confirmed using RAPIDEC^®^ CARBA NP (bioMerieux, Marcy l’Etoile, France) according to the CLSI recommendations [49]. A genotypic characterization was performed for the MDRKp and MDRPa clinical isolates. The presence of carbapenemase genes, such as *bla_KPC_*, *bla_VIM_*, *bla_IMP_*, *bla_NDM_*, and *bla_OXA-48_*, was confirmed using the automated rapid real-time PCR assay BD MAX Check-Points CPO (Check-Points, Wageningen, The Netherlands) on the BD MAX^TM^ system (Ref. 278102, Becton, Dickinson and Company, Franklin Lakes, NJ, USA). *E. coli* ATCC^®^ 25922^TM^, *P. aeruginosa* ATCC^®^ 27853™, and *S. aureus* ATCC^®^ 25923™ strains were obtained from the American Type Culture Collection (ATCC, Manassas, VA, USA) and used as reference.

### 3.3. Disinfection Efficacy Test

An 8% HP solution combined with 30 mg/L silver ions (HP + Ag) and stabilized with 0.03% polyethylene glycol was used for this study. First, the antibacterial activity of HP + Ag was determined by evaluating the MIC as described in the United States Pharmacopeia (USP) Chapter 39 <1072> [51] and the AOAC^®^ 960.09 method [52]. The disinfection efficacy of HP + Ag was assessed against *E. coli* ATCC^®^ 25922^TM^, *P. aeruginosa* ATCC^®^ 27853™, *S. aureus* ATCC^®^ 25923™, and the MDR clinical isolates (MDRKp, MDRPa, and MRSA). To this end, all ATCC strains and clinical isolates were plated on tryptic soy agar (TSA) (Oxoid, CM0131) and incubated at 37 °C for 18–24 h. One colony from each pure culture was then resuspended in 0.85% saline solution to a turbidity value of 0.5 McFarland units, thus containing approximately 1–5 × 10^8^ colony forming units (CFU)/mL. Subsequently, each bacterial inoculum was incubated with different concentrations of HP + Ag. The highest concentration tested was 11,600 mg/L and it was used to perform 1:2 serial dilutions. The inocula were incubated with HP + Ag in a final volume of 10 mL at 37 °C for 18–20 h. A control without HP + Ag was used for each isolate evaluated. The MIC value of HP + Ag for each strain was defined as the lowest concentration that inhibited visible bacterial growth after incubation [52,53]. MICs were determined in duplicate and at least three independent assays were performed for each isolate. A 5% hypochlorite solution was used as the control. The minimum bactericidal concentrations (MBCs) of HP + Ag were determined by pouring the contents of the first three tubes without visible bacterial growth, as obtained during the determination of MICs, on TSA (CM0131, Oxoid Limited, Basingstoke, UK) plates with lecithin and polysorbate. The pour-plate method was used and the plates were incubated at 37 °C for 18–24 h.

### 3.4. Disinfectant Activity on Bacteria in Suspension

The disinfectant activity was evaluated after 1, 5, 10, 15, 20, and 30 min of contact. Based on the disinfection efficacy results, the MIC of 1450 mg/L was used for the activity test on bacteria in suspension. This concentration was the median MIC for all ATCC strains and clinical isolates. The efficacy of HP + Ag in suspension was determined according to the AOAC^®^ 960.09 and 961.02 methods [52,54]. Accordingly, all ATCC strains and resistant clinical isolates were plated on TSA (CM0131, Oxoid Limited, Basingstoke, UK) and incubated at 37 °C for 18–24 h. One colony from each pure culture was then resuspended in 0.85% saline solution to a turbidity value of 0.5 McFarland units, thus containing approximately 1–5 × 10^8^ CFU/mL. Using this initial suspension, dilutions were made for each resistant clinical isolate in 0.85% saline solution to a final concentration of 1–5 × 10^6^ CFU/mL. In addition, a bacterial mixture *of E. coli* ATCC^®^ 25922™, *P. aeruginosa* ATCC^®^ 27853™, and *S. aureus* ATCC^®^ 25923™ was prepared (1–5 × 10^6^ CFU/mL). All bacterial solutions were incubated with 1450 mg/L HP + Ag and organic matter (1% skim milk powder; LP0031, Oxoid Limited, Basingstoke, UK) for 30 min. After 1, 5, 10, 15, 20, and 30 min of incubation, 1 mL of each mixture was seeded on TSA with lecithin and polysorbate using the pour-plate method and incubated at 37 °C for 18–24 h. A 5% hypochlorite solution with a concentration of 2500 mg/L was used as the control. Efficacy in suspension was determined in duplicate and at least three independent assays were performed for each bacterial mixture.

### 3.5. Disinfectant Activity on Surfaces

The efficacy test on surfaces was performed according to the AOAC^®^ 961.02 method [54]. The antibacterial efficacy of the disinfectant was tested on two sterile stainless steel surfaces (20 cm^2^), which were contaminated using two bacterial mixtures: one containing the ATCC strains and the other containing the resistant clinical isolates. For the preparation of bacterial mixtures, separate pure cultures were initially obtained for each strain in TSA and incubated at 37 °C for 18–24 h. One colony from each culture was then resuspended in 0.85% saline solution to obtain initial inocula of 1–5 × 10^8^ CFU/mL. Subsequently, two bacterial mixtures were prepared in 0.85% saline solution using the initial inocula: one containing *E. coli* ATCC^®^ 25922™, *P. aeruginosa* ATCC^®^ 27853™, and *S. aureus* ATCC^®^ 25923™ and another containing the resistant clinical isolates (MDRKp, MDRPa, and MRSA). The resulting mixtures contained 1–5 × 10^3^ CFU/mL. To test the activity on surfaces, 8% VHP combined with 30 mg/L silver ions (VHP + Ag) was applied using the HyperDryMist^®^ automated vaporization equipment. First, each 20 cm^2^ stainless steel surface was inoculated with 10 mL of one bacterial mixture and allowed to air dry at room temperature for 30 min. Subsequently, VHP + Ag was applied to the surfaces and allowed to act for 30 min. Before and after disinfection, samples were collected from the inoculated surfaces using swabs that were subsequently immersed in 10 mL of BHI broth (CM1135, Oxoid Limited, Basingstoke, UK. From these suspensions, samples were seeded on TSA (CM0131, Oxoid Limited, Basingstoke, UK) with lecithin and polysorbate using the pour-plate method and incubated at 37 °C for 18–24 h. All tests were performed in duplicate, with at least three independent assays for each isolate. Finally, to calculate the removal percentage of VHP, the surface disinfection efficacy was determined using the following equation:

(1) $$efficacy\;\left(\%\right)=\;\left(\frac{N_{\theta \;-N_{f}}}{N_{\theta }}\right)\;\times \;100$$

where *N_θ_* and *N_f_* represent the number of microorganisms before and after disinfection, respectively [55]. According to USP Chapter 39 <1072> [51], a disinfectant with an efficacy of >97% is considered effective.

### 3.6. Statistical Analysis

The results were analyzed using descriptive statistical tools with the median. Significant differences in MICs for bacterial strains and times used in the disinfectant activity tests on bacteria in suspension were analyzed and compared using the Kruskal–Wallis nonparametric test, with a significance level of 0.05. All analyses were performed using the statistical software package Stata 14.

## 4. Conclusions

Our findings demonstrated that a solution containing 8% HP and 30 mg/L silver ions has a strong in vitro bactericidal activity against susceptible laboratory strains, resistant clinical isolates, and MDR *K. pneumoniae*, *P. aeruginosa*, and *S. aureus.* Significant differences in disinfectant efficacy were observed between the clinical isolates MDRKp and MDRPa, possibly because of phenotypic and genotypic characteristics that regulate their mechanisms of resistance to conventional antibiotics, which may modulate the efficacy of HP + Ag. A strong in vitro bactericidal activity of HP + Ag was observed against resistant clinical isolates during short periods of exposure. Although no significant differences were found, during the first 15 min of exposure HP + Ag exhibited higher antibacterial activity than 5% sodium hypochlorite, a disinfectant widely used to reduce the transmission of MDR strains in healthcare facilities. Finally, 8% VHP combined with 30 mg/L silver ions was highly effective in reducing the bacterial load on surfaces, with 100% efficacy for ATCC strains and 99.81% for the clinical isolates MDRKp, MDRPa, and MRSA. Based on these results, HP + Ag has the potential to be used as an effective disinfectant to decontaminate surfaces in hospital settings suspected of contamination with MDR bacteria. Further studies should evaluate different types of surfaces and conduct experimental tests directly in hospital facilities.

## Figures and Tables

**Table 1:** Antimicrobial susceptibility and resistance profiles of multidrug-resistant strains of *K. pneumoniae* and *P. aeruginosa*.

Antibiotics, and Resistance Genes and Mechanisms	Strains/MIC (μg/mL)/Interpretative Categories							
	MDRKp1	MDRKp2	MDRKp3	MDRPa1	MDRPa2	MDRPa3	Ec ATCC 25922	Pa ATCC 27853
Ampicillin/sulbactam ^1^	≥32/R	≥32/R	≥32/R	–	–	–	≤2/S	–
Piperacillin/tazobactam ^1^	≥128/R	≥128/R	≥128/R	≥128/R	≥128/R	≥128/R	≤4/S	≤4/S
Cefoxitin ^2^	16/R	≥64/R	32/R	–	–	–	≤4/S	–
Ceftazidime ^3^	≥64/R	≥64/R	≥64/R	32/R	≥64/R	≥64/R	≤1/S	≤1/S
Ceftriaxone ^3^	≥64/R	32/R	≥64/R	–	–	–	≤1/S	–
Cefepime ^3^	≥64/R	≥64/R	32/R	32/R	≥64/R	16/I	≤1/S	≤1/S
Doripenem ^4^	≥8/R	≥8/R	≥8/R	≥8/R	4/I	≥8/R	≤0.12/S	0.25/S
Ertapenem ^4^	≥64/R	4/R	≥8/R	–	–	–	≤0.5/S	–
Imipenem ^4^	8/R	8/R	≥16/R	≥16/R	1/S	≥16/R	≤0.25/S	2/S
Meropenem ^4^	16/R	≥16/R	≥16/R	8/R	4/I	8/R	≤0.25/S	≤0.25/S
Amikacin ^5^	≥64/R	16/S	≥64/R	≥64/R	≥64/R	≥64/R	≤2/S	≤2/S
Gentamicin ^5^	4/S	16/R	4/S	4/S	≥16/R	4/S	≤1/S	≤1/S
Ciprofloxacin ^6^	≥4/R	≥4/R	≥4/R	≥4/R	≥4/R	2/R	≤0.25/S	≤0.25/S
Colistin ^7^	–	–	–	≥16/R	≤0.5/S	≥16/R	–	≤0.5/S
Carba NP result	POS	POS	POS	POS	POS	POS	NEG	NEG
EDTA/SMA synergy test	NEG	NEG	NEG	POS	POS	POS	NEG	NEG
Boronic acid synergy test	POS	POS	POS	–	–	–	NEG	NEG
Carbapenemase genes								
*bla_KPC_*	POS	POS	POS	NEG	NEG	POS	NEG	NEG
*bla_VIM_* and/or *bla_IMP_*	NEG	NEG	NEG	POS	POS	POS	NEG	NEG
*bla_NDM_*	NEG	NEG	NEG	NEG	NEG	POS	NEG	NEG
*bla_OXA-48_*	NEG	NEG	NEG	NEG	NEG	NEG	NEG	NEG

Abbreviations: MIC, minimal inhibitory concentration; MDRKp, Multidrug-Resistant *K. pneumoniae*; MDRPa, Multidrug-Resistant *P. aeruginosa*; Ec, *Escherichia coli*; Pa, *P. aeruginosa*; R, resistant; I, intermediate; S, susceptible; NEG, negative; POS, positive. ^1^ Penicillin + β-lactamase inhibitors; ^2^ cephamycins; ^3^ extended-spectrum cephalosporins; ^4^ carbapenems; ^5^ aminoglycosides; ^6^ fluoroquinolones; ^7^ polymyxins. Dashes indicate that antibiotics and resistance genes and/or mechanisms were not determined.

**Table 2:** Antimicrobial susceptibility and resistance profiles of methicillin-resistant *S. aureus* strains.

Strain	MIC (μg/mL) for Antibiotic/Interpretative Category																
	AMP ^1^	CIP ^2^	CLI ^3^	ERY ^4^	GEN ^5^	LVX ^2^	LZD ^6^	MIN ^7^	MXF ^2^	NIT ^8^	OXA ^9^	Q-D ^10^	RIF ^11^	TEC ^12^	TET ^7^	SXT ^13^	VAN ^12^
MRSA1	0.5/S	≤0.5/S	≤0.25/S	≥8/R	≤0.5/S	≤0.12/S	2/S	≤0.5/S	≤0.25/S	≤16/S	≥4/R	≤0.25/S	≤0.5/S	≤0.5/S	≤1/S	≤10/S	1/S
MRSA2	0.5/S	≤0.5/S	≤0.25/S	≤0.25/S	≤0.5/S	≤0.12/S	2/S	≤0.5/S	≤0.25/S	≤16/S	≥4/R	≤0.25/S	≤0.5/S	≤0.5/S	≤1/S	≤10/S	1/S
MRSA3	0.5/S	≤0.5/S	≤0.25/S	≤0.25/S	≤0.5/S	≤0.12/S	2/S	≤0.5/S	≤0.25/S	≤16/S	≥4/R	≤0.25/S	≤0.5/S	≤0.5/S	≤1/S	≤10/S	1/S
Sa ATCC 29213	0.5/S	≤0.5/S	≤0.25/S	≤0.25/S	≤0.5/S	≤0.12/S	2/S	≤0.5/S	≤0.25/S	≤16/S	≤0.25/S	≤0.25/S	≤0.5/S	≤0.5/S	≤1/S	≤10/S	≤0.5/S

Abbreviations: MIC, minimal inhibitory concentration; MRSA, methicillin-resistant *S. aureus*; Sa, *S. aureus*; AMP, ampicillin; CIP, ciprofloxacin; CLI, clindamycin; ERY, erythromycin; GE, gentamicin; LVX, levofloxacin; LZ, linezolid; MIN, minocycline; MXF, moxifloxacin; NIT, nitrofurantoin; OXA, oxacillin; Q-D, quinupristina/dalfopristina; RIF, rifampicin; TEC, teicoplanin; TET, tetracycline; SXT, trimethoprim/sulfamethoxazole; VAN, vancomycin; R, resistant; S, susceptible; ^1^ penicillin; ^2^ fluoroquinolones; ^3^ lincosamides; ^4^ macrolides; ^5^ aminoglycosides; ^6^ oxazolidinones; ^7^ tetracyclines; ^8^ nitrofurans; ^9^ anti-staphylococcal b-lactams (or cephamycins); ^10^ streptogramins; ^11^ ansamycins; ^12^ glycopeptide; ^13^ folate pathway inhibitors.

**Table 3:** In vitro antibacterial activity of HP + Ag against resistant clinical isolates of *K. pneumoniae*, *P. aeruginosa*, and *S. aureus*, and ATCC strains.

Strains (n)	PH+Ag		Control (Hypochlorite 5%)		*p*-Value
	MIC (mg/L)	MBC (mg/L)	MIC (mg/L)	MBC (mg/L)	
MDRKp (3)	362.5–725	362.5–725	2500–5000	2500–5000	0.04
MDRPa (3)	1450–5800	1450–5800	2500–5000	2500–5000	0.51
MRSA (3)	725–1450	725–1450	5000	5000	0.04
Ec ATCC 25922	725–1450	725–1450	1250	1250	0.99
Pa ATCC 27853	1450–2900	1450–2900	5000	5000	0.02
Sa ATCC 25923	362.5	362.5	3750–5000	3750–5000	0.02

**Table 4:** In vitro efficacy of 8% VHP combined with 30 mg/L silver ions against sets of ATCC strains and clinical isolates on surfaces.

Strains	Bacterial Count Prior to HPV Exposure		Bacterial Count after HPV Exposure		Efficacy (%)
	CFU	Log_10_ Reduction	CFU	Log_10_ Reduction	
Set of ATCC strains	9.39 × 10^2^	2.97	0.00	0.48	100
Set of resistant clinical isolates	1.65 × 10^3^	3.22	3.00	0.00	99.81

## Data Availability

Not applicable.
